# What Understanding Is For

**DOI:** 10.1007/s42113-026-00300-z

**Published:** 2026-04-14

**Authors:** Benjamin A. Motz, Joseph P. Magliano, Kathryn S. McCarthy, Danielle S. McNamara

**Affiliations:** 1 Department of Psychological and Brain Sciences, Cognitive Science Program, Indiana University, Bloomington, Indiana USA; 2 Department of Learning Sciences, Georgia State University, Atlanta, Georgia USA; 3 Learning Engineering Institute, Department of Psychology, Arizona State University, Tempe, Arizona USA

## Abstract

Rendering understanding as a general causal account is problematic. This leads us toward accusations of partial understanding, or illusory understanding, characterized by understanding that lacks depth. The discourse comprehension literature offers an alternative: rendering understanding as the construction of a situation model, a model that integrates knowledge, purpose, and context. On this account, the question is never whether understanding is complete, but whether it is adequate for what it aims to achieve.

Is science under a cloud of illusions? Shiffrin et al. (2026) suggest that it is, arguing that scientists often mistake partial causal accounts for genuine understanding. The authors acknowledge the fuzziness of “understanding” at the outset, but then proceed to define it as causal explanation, and with that standard in hand, they document the many ways scientific understanding falls short. Comprehension researchers start from a different premise. Rather than setting the fuzziness aside, they make it the object of empirical inquiry. What that inquiry reveals is that understanding is not a scalar quantity but a cognitive construction shaped by situations and goals. The adequacy of any understanding is most meaningfully evaluated relative to the purpose it was constructed to serve (Britt et al., 2018; Graesser et al., 1994).

Kintsch’s Construction-Integration model (1988, 1998) was a seminal theoretical framework that helped move the field away from overly simplistic questions about comprehension. The CI model described the cognitive processes that give rise to situation models, which are probabilistic outcomes of those processes. Crucially, an expert constructs more elaborate understanding than a novice, but that richness is relative to the situation in focus, not an absolute measure of proximity to some complete causal account. Depth, in other words, is a property of fit between a representation and a purpose, not an amount of scientific completeness.

This was not merely a choice to shift the goalposts. It was an empirical conclusion, arrived at through decades of scientific effort. Consider a central debate in discourse psychology from the early 1990s. Researchers asked questions like “What inferences are necessary for comprehension?” as though those questions had universal and determinate answers (e.g., Magliano et al., 1993; McKoon & Ratcliff, 1992). This is precisely the kind of framing that Shiffrin and colleagues caution against — a question posed as though a complete explanatory answer is within reach. Such a question turns out to be underspecified. Necessary for what purpose? Adequate for whose goal? The move was not a retreat from causal explanation, but a reckoning with what causal explanation in a complex domain requires.

This history matters because it is precisely the humble reckoning Shiffrin, Stigler, and Keil are calling for in science generally. The authors are right that illusions of understanding are pervasive and costly. Comprehension research faced the same reckoning and found its way through, by replacing a flawed conception of understanding with one that situates evaluation in purpose and context.

The question, then, is not “does the statistician understand regression?” but rather, “does the statistician understand what regression delivers under these assumptions for this purpose?” This shift dissolves the illusion of a single, context-independent standard against which understanding can be declared partial and wanting.

There is an irony worth naming here. Shiffrin, Stigler, and Keil take issue with scientists who reify their abstractions, who treat models as complete explanations. Yet “understanding” moves through their essay as though it were a natural kind with a determinate depth dimension, measured against the ideal of causal completeness. In other words, the authors critique scientists for reifying abstractions while reifying their own abstraction of what understanding is. In fairness, Shiffrin and colleagues acknowledge the fuzziness, and acknowledge that any phenomenon has multiple causes, but then march ahead with accusations of “partial understanding.” Those accusations deserve scrutiny. If “partial understanding” refers to a mismatch between representational scope and inferential purpose, then the accusation is fair and the remedy is clear. But if “partial understanding” means a failure to achieve causal completeness, it is an accusation no scientist could ever escape, because causal completeness is not a standard any cognitive system could achieve. In advancing that second, fuzzier sense of the term as though it were the consensus goal of science, the illusions being diagnosed are partly of the authors’ own making.

How would the comprehension researcher define understanding? First, they would bristle, but if pressed, they might offer that understanding is more like a verb than a noun, a process rather than a possession, and one which can take many possible forms depending on who is doing the understanding, what they already know, and what they need the understanding to accomplish. Kintsch (1998) described comprehension as the construction of a situation model: a mental representation that goes beyond the explicit content of a text to incorporate inference, background knowledge, and context. Of course, situation models vary along many dimensions, and a model that integrates propositions into a well-connected, elaborate, internally consistent representation may be more adequate and useful than one that leaves contradictions unresolved or connections unmade. But the quality of construction is evaluated relative to a situation and purpose. What cannot be assumed is that there is a single purpose-independent dimension along which all situation models can be ordered, and then evaluated against the ideal of causal completeness.

If understanding is a construction rather than a depth, then science does not advance by eliminating simplification (sinking into ever-deepening complexity). It advances by aligning understanding with purpose. Four scientists studying monarch butterfly navigation from different angles are not all falling short of one complete causal account. They are building different structures for different purposes, and those structures can coexist productively without collapsing into one another (Marr, 1992; Poggio, 2012). Similarly, Jonas and Kording (2017) demonstrated that complete mechanistic access to a microprocessor did not yield a satisfying account of it, because understanding depends on the question being asked. Completeness is not the standard. Functional adequacy for a specified purpose is.

A scientist who reads Shiffrin, Stigler, and Keil’s essay and feels existential dread is accepting a standard they were never obligated to accept. The dread comes from measuring one’s constructions against a standard that no cognitive system could have evolved to achieve. Comprehension research offers a better framework: one that asks what understanding is for, and evaluates it accordingly.

## Data Availability

No datasets were generated or analysed during the current study.
